# Supplementary material for: Tobacco Mosaic Virus Infection of Chrysanthemums in Thailand: Development of Colorimetric Reverse-Transcription Loop-Mediated Isothermal Amplification (RT–LAMP) Technique for Sensitive and Rapid Detection
Source: Plants (Basel). 2022 Jul 6;11(14):1788. doi: 10.3390/plants11141788 (PMC9325109; doi:10.3390/plants11141788)
Supplement: Supplementary file 1 [file plants-11-01788-s001.zip › plants-1743044-supplementary.pdf]

Supplementary Figure

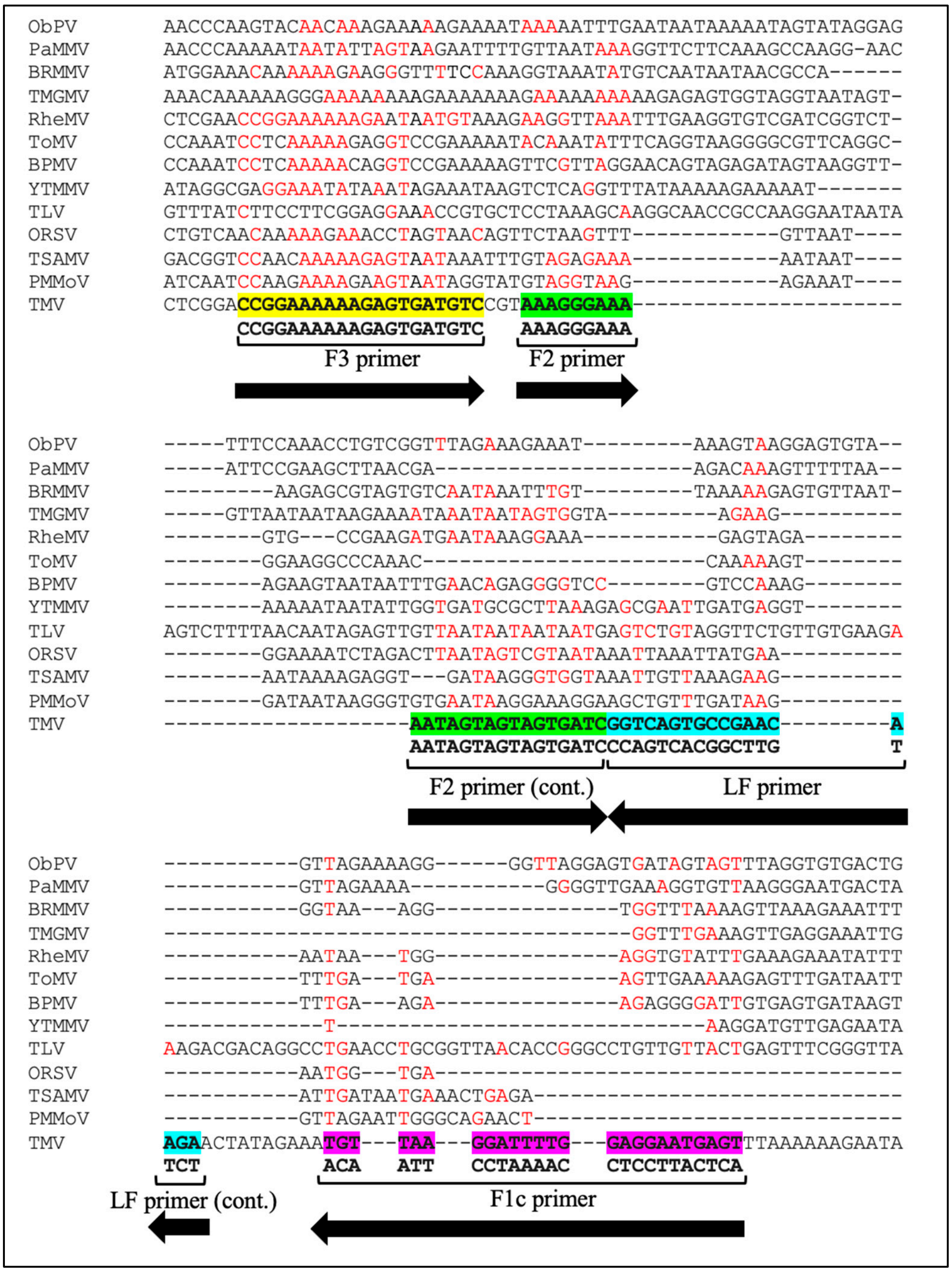

Supplementary figure S1. *Cont.*

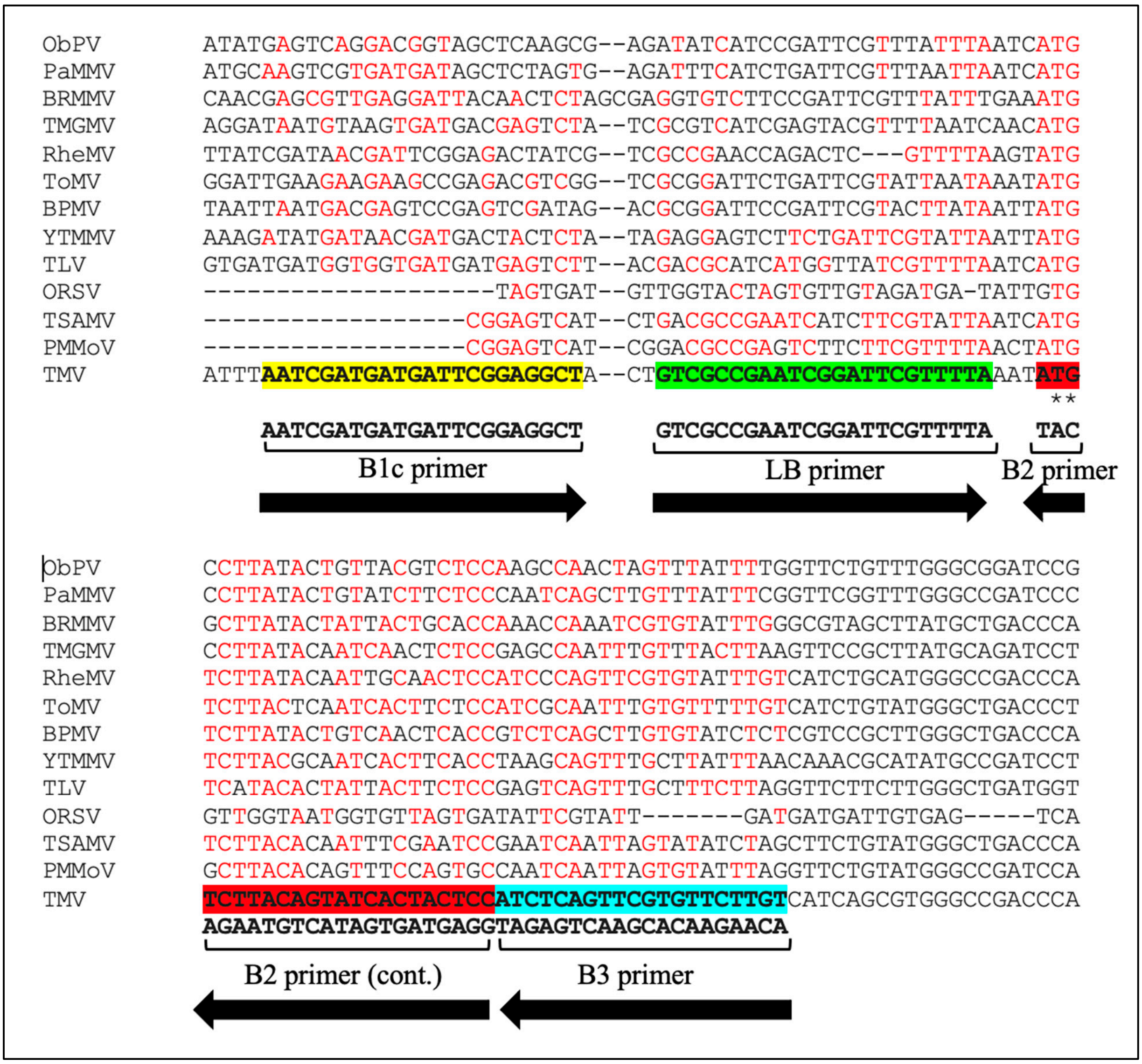

Supplementary figure S1. Multiple alignments of partial *CP* gene sequences of Solanaceae-infecting tobamoviruses with the binding sites of LAMP primers. The alignments were made using partial *CP* gene sequences of Solanaceae-infected tobamoviruses described in the main text and the Clustal Omega (https://www.ebi.ac.uk/Tools/msa/clustalo/) with default settings of parameters. The LAMP primers' binding sites in TMV sequences are highlighted. In other tobamoviruses, residues identical to the TMV sequence are shown in red. Asterisks denote residues identical in all the tobamoviruses examined.
